# Albumin Homodimers in Patients with Cirrhosis: Clinical and Prognostic Relevance of a Novel Identified Structural Alteration of the Molecule

**DOI:** 10.1038/srep35987

**Published:** 2016-10-26

**Authors:** Maurizio Baldassarre, Marco Domenicali, Marina Naldi, Maristella Laggetta, Ferdinando A. Giannone, Maurizio Biselli, Daniela Patrono, Carlo Bertucci, Mauro Bernardi, Paolo Caraceni

**Affiliations:** 1 Department of Medical and Surgical Sciences, Alma Mater Studiorum University of Bologna, Italy; 2 Center for Applied Biomedical Research (C.R.B.A.), Alma Mater Studiorum University of Bologna, Italy; 3 Department of Pharmacology and Biotechnology, Alma Mater Studiorum University of Bologna, Italy; 4 Centralized Laboratory, S. Orsola-Malpighi University Hospital, Bologna, Italy

## Abstract

Decompensated cirrhosis is associated to extensive post-transcriptional changes of human albumin (HA). This study aims to characterize the occurrence of HA homodimerization in a large cohort of patients with decompensated cirrhosis and to evaluate its association with clinical features and prognosis. HA monomeric and dimeric isoforms were identified in peripheral blood by using a HPLC-ESI-MS technique in 123 cirrhotic patients hospitalized for acute decompensation and 50 age- and sex-comparable healthy controls. Clinical and biochemical parameters were recorded and patients followed up to one year. Among the monomeric isoforms identified, the N- and C-terminal truncated and the native HA underwent homodimerization. All three homodimers were significantly more abundant in patients with cirrhosis, acute-on-chronic liver failure and correlate with the prognostic scores. The homodimeric N-terminal truncated isoform was independently associated to disease complications and was able to stratify 1-year survival. As a result of all these changes, the monomeric native HA was significantly decreased in patients with cirrhosis, being also associated with a poorer prognosis. In conclusion homodimerization is a novel described structural alteration of the HA molecule in decompensated cirrhosis and contributes to the progressive reduction of the monomeric native HA, the only isoform provided of structural and functional integrity.

The human albumin (HA) molecule presents both quantitative and qualitative changes in patients with decompensated cirrhosis^1^. With the progression of the disease, HA not only undergoes a reduction of its plasma concentration, but also develops several structural alterations^2^. As the biological properties of HA are closely related to the preservation of its structural integrity^3^, these post-transcriptional changes could likely impair many non-oncotic functions of the molecule, such as binding and transport, detoxification, free radical scavenging, and others^3^.

The most likely cause of the HA damage is represented by the pro-inflammatory and pro-oxidant intra-vascular milieu that characterizes the advanced stages of cirrhosis^4^,^5^ as well as of other chronic systemic diseases^6^. Indeed, several *in vitro* studies have shown that the exposure to various oxidant agents can produce a series of structural damages, most of which are localized at the cysteine residue at position 34 (Cys-34), the main functional site of the entire molecule^3^,^7^.

Besides the well-established reversible and irreversible oxidation of the Cys-34^5^,^8^,^9^, cirrhosis is characterized by a diffuse structural damage that involves several other sites of the HA molecule and worsens in parallel with the severity of the disease^2^. Using an analytical approach based on liquid chromatography tandem mass spectrometry (LC-MS), we have identified several altered HA isoforms deriving from reversible or irreversible oxidation of the Cys-34 residue, truncation of the N-terminal and C-terminal portion, and glycosylation that can occur alone or in various combination^2^. Due to the accumulation of these structurally abnormal molecules, the relative amount of HA in its native form, characterized by a fully preserved structure and function, is substantially reduced in patients with advanced cirrhosis. Interestingly, the native HA is a potent predictor of 1-year survival in patients with cirrhosis, carrying a far more accurate prognostic power than the serum albumin concentration as routinely measured in clinical practice^2^.

Homodimerization consists in the formation of a chemical compound by two identical molecules which can lead to a functional loss with respect to the monomeric molecules^10^. Although it is known from *in vitro* studies that HA can form homodimers as a result of oxidative stress^7^, the evidence that the homodimerization process occurs also in clinical diseases is limited to sporadic observations in patients undergoing hemodialysis^11^ or with liver cirrhosis^7^.

With this in mind, as part of a multidisciplinary research project aimed at the evaluation of the structural and functional alterations of HA in patients with decompensated cirrhosis^2^,^12^, the present study has been designed to determine the extent and relevance of HA homodimerization in a large cohort of patients with cirrhosis admitted to the hospital because of an acute decompensation (AD) of the disease. To achieve this aim, we assessed whether in patients with cirrhosis HA homodimers: (1) are increased as compared to healthy controls, thus contributing to the reduction of the circulating amount of the native HA; (2) are associated with the severity of cirrhosis or the occurrence of complications of the disease; and, (3) predict patient prognosis.

## Materials and Methods

### Patients

From July 2011 to March 2012 all the patients with cirrhosis admitted to our Department because of the onset of a complication were screened for the study enrolment. The diagnosis of cirrhosis was based on clinical, biochemical, ultrasound, and endoscopic features. Exclusion criteria were: age under 18 years, admission for a scheduled procedure, hepatocellular carcinoma (HCC) exceeding the Milan criteria^13^, heart and respiratory failure, organic renal diseases, onco-hematologic disorders, protein-losing syndromes, albumin infusion in the previous month and ongoing immunosuppressive treatment. Fifty healthy voluntaries comparable with patients for age- and sex were enrolled as reference population.

Informed written consent was obtained from patients and healthy controls, the study protocol was approved by the S.Orsola Malpighi University Hospital ethical committee and all experimental methods involving human participants were in accordance with the 1975 Declaration of Helsinki.

### Study design and definitions

Patients were enrolled within 24 h from admission. At that time, peripheral blood was withdrawn from the brachial vein into pyrogen-free tubes (Vacutainer EDTA tubes, Becton Dickinson Italia, Italy). Blood samples were immediately centrifuged at 3,000 g × 10 minutes and plasma was aliquoted into cryotubes (Corning Inc., Corning BV, Netherlands) and stored at −80 °C until analysis. A routine biochemical evaluation, including liver and renal function tests and coagulation parameters was performed, while the Model of End-stage Liver Disease (MELD)^14^, Child-Pugh^15^, CLIF Consortium organ failure score (CLIF-C OFs)^16^ and the CLIF Consortium acute decompensation score (CLIF-C ADs)^17^ were calculated to assess disease severity. In each patient the presence and the grade of Acute-on-Chronic Liver Failure (ACLF) was also evaluated.

Ascites was clinically evident or was ascertained by ultrasound examination; hepatic encephalopathy (HE) was graded according to the West-Haven classification^18^; renal impairment was defined as a serum creatinine greater than 1.5 mg/dL; upper gastrointestinal bleeding was confirmed by endoscopy. Bacterial infections were diagnosed as follows: (a) spontaneous bacterial peritonitis: polymorphonuclear cell count in ascites > 250/mm^3^; (b) urinary infection: positive culture or >10 leukocytes per high-power field in urine associated to suggestive clinical symptoms and signs; (c) pneumonia: presence of new infiltrates on chest x-ray; (d) skin/soft tissue infection: physical exam findings of swelling, erythema, heat and tenderness in the skin; (e) spontaneous bacteremia: positive blood cultures and no evident cause of bacteremia; (f) other infections (diverticulitis, colecystitis, meningoencephalytis): according to specific findings at the laboratory, microbiological, and imaging assessment. Diabetes was diagnosed according to the American Diabetes Association guidelines^19^. Patient survival was recorded during hospitalization and after discharge up to 1 year.

### Assessment of the HA isoforms

#### *HPLC*/*ESI-MS measurements*

Plasma samples were diluted 1:10 with water/acetonitrile (98/2) (v/v) mixture and filtered with 0.22 μm filter (Merck KGaA, Darmstadt, Germany). A further dilution (1:100) with the same mixture was then performed before each injection.

Nano-LC-nano-ESI-QTOF analysis was carried out by using a nano-LC Agilent 1100 Series (Walbronn, Germany) interfaced with a Q-TOF hybrid analyser (Q-TOF Micro, Micromass, Manchester, UK) with nano-Z-spray ion source. Reversed phase chromatographic separations of HSA from other plasma proteins was performed on a C8 (50 mm × 75 μm; 3.5 μm) column, using an elution gradient from A [water: acetonitrile: FA (99:1:0.1) (v/v)]/B [acetonitrile: water: FA (98:2:0.1) (v/v)] 85/15 (v/v), to A/B 20/80 (v/v), in 20 min, at a flow rate of 0.5 μL/min; system was equipped with an auto-sampler and the injection volume was 1 μL. The column was equilibrated with the mobile phase composition of the starting conditions for 10 min before the next injection.

The ESI-QTOF source temperature was set at 100 °C, the capillary voltage at 3.5 kV, and the cone voltage at 42 V. The scan time was set at 2.4 sec and the inter scan time at 0.1 sec. The mass chromatograms were recorded as total ion current (TIC), within 600 and 2,500 m/z (mass/charge). To characterize HA isoforms by molecular weight determination, multicharged mass spectra were acquired on the peak apex identified as HSA by Q-TOF. Deconvoluted ESI mass spectra of HA were obtained by using MassLynks 4.1 software. The peak averaged mass spectra were reconstructed and the mass of the HSA isoforms derived.

#### *HA isoforms identification and relative quantification*

In addition to the native HA, six HA isoforms were identified by analyzing the 65.800–67.100 Da mass range, as previously reported^20^. The homodimeric isoforms were identified by extending the analysis from 61.500 to 138.000 mass range.

The relative abundance of each homodimeric HA isoform and that of the corresponding monomeric isoform was expressed as percentage of relative amount of the isoforms over total isoforms evaluated according to the following formula:


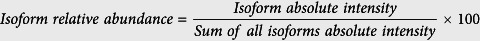


### *In vitro* dose-dependent oxidative experiments

Dose-dependent oxidative experiments were performed by exposing plasma samples from a healthy subject to increasing concentration of tert-Butyl hydroperoxide (t-BuOOH). Briefly, t-BuOOH (Sigma–Aldrich, USA) was added to plasma samples to reach the final concentration of 0.1, 0.5 or 1 mM. Samples not treated with t-BuOOH were used to determine basal HA isoforms relative abundance. Each mixture was then incubated at 37 °C for 24 hours and, afterwards, analyzed by means of LC-MS according to the method described above.

### Statistical analysis

Variables were expressed as mean and standard deviation or frequencies according to their distribution. The relationship between relative abundance of HA isoforms and clinical scores was evaluated with the Spearman’s rho correlation analysis.

The association between HA isoforms relative amount and specific complication of cirrhosis was assessed by means of Student t-test while one-way analysis of variance (ANOVA) was used to compare the HA isoforms relative abundance in *in vitro* experiments. The ANOVA analysis was also used to compare the relative amount of HA homodimers between patients without ACLF, with grade 1 ACLF or with grade 2/3 ACLF. Binary logistic regression was used as multivariate model. Only variables showing a p value < 0.05 at the univariate analysis were tested for association to the specific complication of the disease in the multivariate analysis.

The association between HA isoforms and 1-year survival was assessed using the Kaplan-Meier method, while intergroup comparisons were performed by the log-rank test. In order to plot survival curves, continuous variables were categorized according to their best cut-off value defined as the level associated to the highest sensitivity and specificity in discriminating 1-year survivors from not survivors at Receiver Operating Characteristics (ROC) curve analysis.

All tests were two sided and values of *p* less than 0.05 were considered statistically significant. The analyses were performed using Statistical Package for Social Sciences v.20.0 software (IBM Corporation, New York, USA).

## Results

### Patients

One hundred twenty-three cirrhotic patients were included in the study. Their age and gender (60 ± 11 years; 83 [67%] males) did not differ from that of the 50 healthy subjects (58 ± 8 years; 30 [60%] males).

Etiology of cirrhosis, causes of hospitalization, hemodynamic and biochemical parameters, and prognostic scores are summarized in Table 1. At admission ACLF was present in 21 cases, namely 15 patients had ACLF grade 1, 5 patients ACLF grade 2 and only 1 patient ACLF grade 3 according to the CLIF consortium criteria^16^.

### HA homodimeric isoforms in patients with cirrhosis and healthy controls

**Figure 1:**
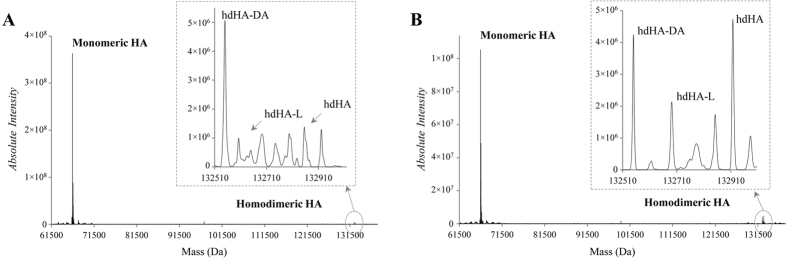
Representative deconvoluted ESI-MS spectra, reporting HA isoforms absolute intensity (count/second) over mass (Da), from a control subject (**A**) and a cirrhotic patient (**B**) with a magnification of the mass range between 132000 and 133000 Da (dotted circle and arrow) where the human albumin (HA) homodimeric isoforms can be detected. hdHA-DA: homodimeric N-terminal truncated isoform; hdHA-L: homodimeric C-terminal truncated isoform; hdHA: homodimeric native isoform.

Three homodimeric HA isoforms were identified based on the expected molecular weight of each HA monomer and dimer. Namely, the HA isoforms undergoing homodimerization were the N-terminal truncated HA (hdHA-DA), characterized by the truncation of the last two amino acidic residues at the N-terminal portion, the C-terminal truncated (hdHA-L), characterized by the truncation of the last aminoacidic residue at the C-terminal portion, and the native unchanged HA isoform (hdHA-native) (Fig. 1).

**Figure 2:**
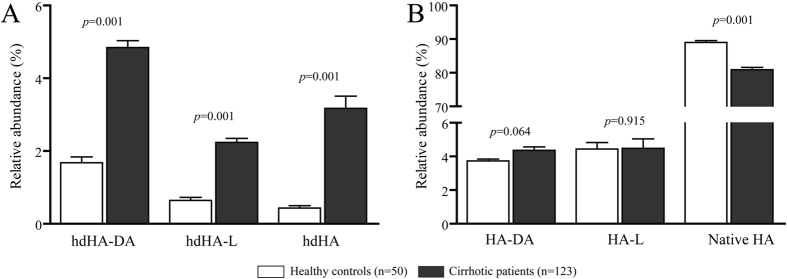
Relative abundance of Human Albumin (HA) homodimers (panel A) and the corresponding monomeric forms (panel B) in healthy controls and patients with cirrhosis. Data is presented as mean and SEM. hdHA-DA: homodimeric N-terminal truncated isoform; hdHA-L: homodimeric C-terminal truncated isoform; hdHA: homodimeric native isoform; HA-DA: monomeric N-terminal truncated isoform; HA-L: monomeric C-terminal truncated isoform; HA: monomeric native isoform.

All these HA homodimeric isoforms were detected in both patients with cirrhosis and healthy controls (Fig. 1), but the extent of homodimerization was significantly greater in patients, being the hdHA-DA isoform, which results from two monomeric isoforms presenting the truncation at the N-terminal site, the most abundant (Fig. 2, panel A).

Consistently with previous results^2^, the amount of the monomeric native HA, the only isoform with a fully preserved structure, was lower in patients than controls, while the monomeric N-terminal (HA-DA) and C-terminal truncated (HA-L) isoforms did not significantly differ (Fig. 2, panel B).

**Figure 3:**
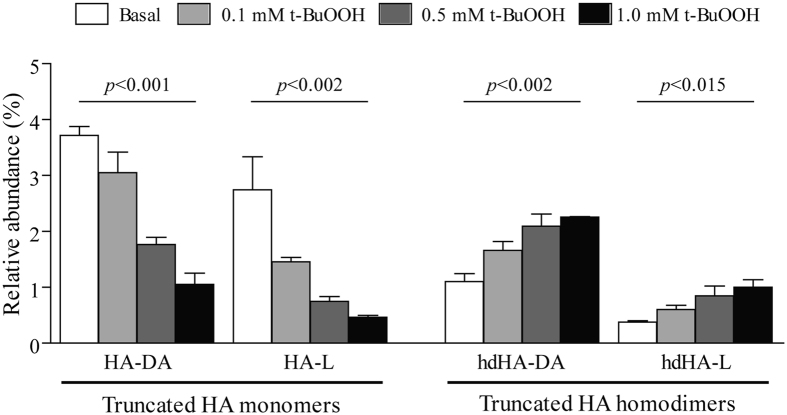
N-terminal and C-terminal truncated monomeric (HA-DA and HA-L) and homodimeric (hdHA-DA and hdHA-L) human albumin (HA) isoforms in the plasma of a healthy individual exposed to increasing concentration of tert-Butyl hydroperoxide (t-BuOOH). For each isoform the mean and SEM of four replicates and the global ANOVA p value are reported.

In order to explain this latter finding, we performed *in vitro* experiments by challenging the plasma from a healthy subject with a pro-oxidant agent. t-BuOOH induced a dose-dependent rise in the relative amount of the homodimeric truncated isoforms with a significant fall in the amount of the corresponding monomers (Fig. 3), suggesting that the lack of increase, in patients with cirrhosis, of the monomeric truncated isoforms results by their tendency to form homodimers in a pro-oxidant environment.

### Relationship between HA homodimeric isoforms and clinical features

All the 3 homodimeric HA isoforms significantly correlated with MELD, Child-Pugh, CLIF-C OF and CLIF-C AD scores, while only the native monomeric HA was significantly and inversely correlated with all prognostic scores (Table 2).

We then evaluated the association with the presence of disease-related complications diagnosed at hospital admission. Ascites, renal impairment, and bacterial infection were associated with specific patterns of HA isoforms. Namely, the homodimeric hdHA-DA isoform was significantly more represented in all patients with complications, irrespective of the type of the clinical manifestation, while the homodimeric hdHA isoform was associated only with the presence of renal impairment. In contrast, the native HA was the unique monomeric isoform to be associated with complications. Indeed, its relative abundance was reduced in all complicated patients reaching the statistical significance in those presenting ascites or renal impairment (Supplementary Table 1).

We finally performed a logistic regression analysis to assess whether the associations found between HA isoforms and specific clinical complications were unrelated to the severity of the underlying disease. Only the homodimeric hdHA-DA isoform was independently associated with the presence of ascites (OR 1.321; CI 95% 1.071–1.630; p = 0.009), renal impairment (OR 1.377; CI 95% 1.084–1.750; p = 0.009), or bacterial infection (OR 1.537; CI 95% 1.229–1.922; p < 0.001), irrespective of MELD or Child-Pugh scores (Supplementary Table 2).

In contrast, the relative amount of HA homodimers did not differ in patients presenting or not variceal bleeding and grade III and IV HE (Supplementary Table 3).

**Figure 4:**
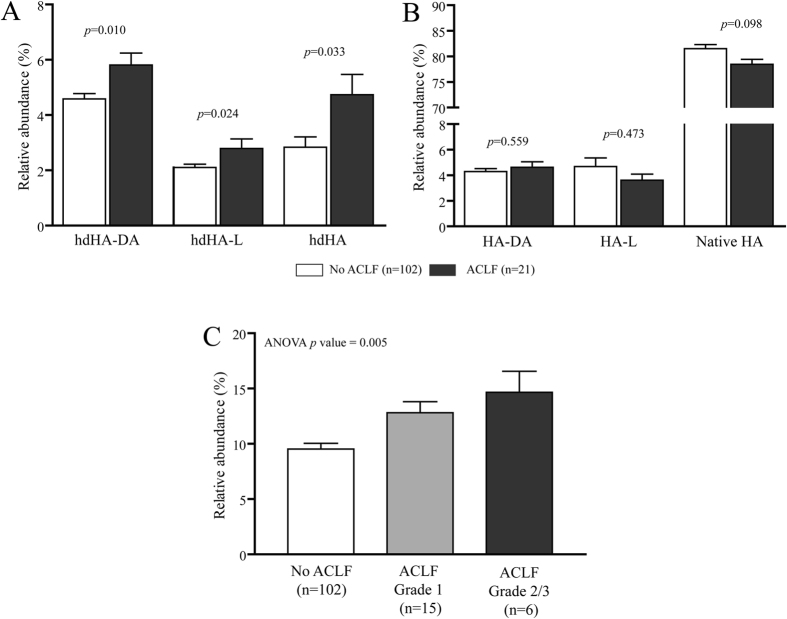
Relative abundance of Human Albumin (HA) homodimers (panel A) and the corresponding monomeric isoforms (panel B) in patients with or without acute-on-chronic liver failure (ACLF) at hospital admission. The relative amount of grouped HA homodimers according to the presence and the grade of ACLF is also reported in panel C. Data is presented as mean and SEM. hdHA-DA: homodimeric N-terminal truncated isoform; hdHA-L: homodimeric C-terminal truncated isoform; hdHA: homodimeric native isoform; HA-DA: monomeric N-terminal truncated isoform; HA-L: monomeric C-terminal truncated isoform; HA: monomeric native isoform.

Finally, we assessed the association between ACLF and homodimeric and monomeric HA isoforms. Although the low number of ACLF cases recorded in our series, we found that all the 3 homodimeric isoforms were significantly more abundant in patients presenting the syndrome at hospital admission than in those without ACLF, while no differences were observed when the monomeric HA isoforms were compared (Fig. 4, panel A and B). Additionally, by grouping the relative amount of the 3 HA homodimeric isoforms, the cumulative amount increased progressively with the grade of ACLF (Fig. 4, panel C).

### HA homodimeric isoforms and 1- year patient survival

**Figure 5:**
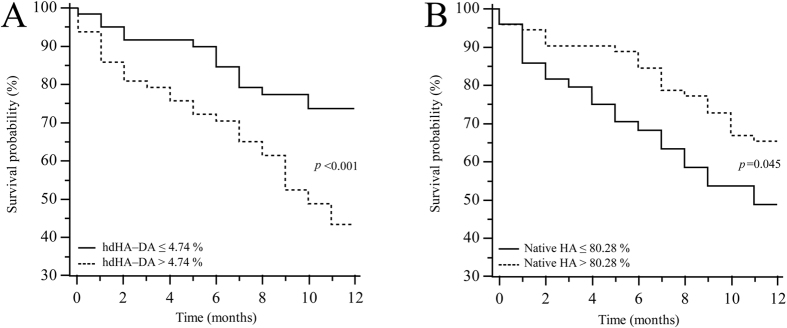
Kaplan-Meier survival curves for the homodimeric N-terminal truncated isoform (hdHA-DA) (panel A) and the native HA isoform (panel B) dichotomized according to their best cut off, as determined by the receiver operating characteristic curve analysis.

After 1 year, 74 patients (61.2%) were alive. Only the homodimeric hdHA-DA and monomeric native HA were able to significantly stratify patient prognosis when categorized according their best cut-off. Namely, patients with a relative amount of the homodimeric hdHA-DA lower or equal than 4.74% showed significantly higher survival than their counterparts (10.22 ± 0.44 vs 8.28 ± 0.56 months, p = 0.001) (Fig. 5, panel A). Finally, patients with a relative amount of monomeric native HA higher than 80.28% had a significant longer survival (9.94 ± 0.42 vs 8.33 ± 0.64 months, p = 0.045). (Fig. 5, panel B).

## Discussion

The present study provides evidence that among the many post-transcriptional abnormalities to which circulating albumin undergoes in patients with cirrhosis^2^,^9^,^21^,^22^,^23^, the development of albumin homodimers has to be added.

Homodimerization consists in the formation of a chemical compound following covalent or non-covalent interaction by two identical molecules^10^. The effects of this process are quite heterogeneous. In some cases, homodimeric proteins are provided of functional gain with respect to the monomeric molecules and are involved in the modulation of enzyme activity, ion channels, receptors, and transcription factors, while, in others, homodimerization leads to the inhibition of the activities carried out by the monomeric molecule^10^.

Although it is known that HA can form homodimers, available data on this matter are limited. *In vitro* studies have demonstrated that this process is often triggered by free radicals and, therefore, HA homodimers have been proposed as marker of oxidative stress^11^. HA homodimers and oligomers were also found in commercial preparations and their absence is considered a parameter of stability of the protein over the shelf life of the formulation^24^. However, the analytical methods employed in these previous reports, namely SDS-PAGE and high performance size exclusion chromatography coupled to UV detector, did not allow the characterization of the homodimeric HA microheterogeneity^11^,^24^. Furthermore, whether HA homodimerization occurs in humans and whether or not this process assumes clinical relevance is not known as yet. Indeed, an increased formation of HA disulfide homodimers by using SDS-PAGE and Western blot analysis has been described in plasma from 3 patients undergoing hemodialysis^11^. Finally, a pilot study has shown very recently the *in vivo* generation of HA homodimers in the plasma of patients with cirrhosis. *In vitro* experiments have then demonstrated that the homodimerization process takes place through the formation of a disulfide bonds between the Cys-34 residue of two HA monomers as a result of oxidative stress^7^.

The present study demonstrates for the first time that homodimerization of the circulating HA commonly occurs in decompensated cirrhosis, thus contributing, along with other molecular abnormalities, to reduce the proportion of the native molecule with fully preserved structure and function.

By using a HPLC-MS analytical approach, which allows to discriminate between isoforms whose molecular weight differs even by few Daltons, we were able to establish that the homodimerization process leads to the generation of three main different isoforms, which were significantly more abundant as compared to the low amounts found in healthy subjects^7^. Interestingly, all these isoforms (hdHA-DA, hdHA-L, hdHA-native) result from the combination of monomers presenting the Cys-34 residue in the reduced state. In fact, the homodimerization process requires the availability of the Cys-34 site free to react with a homologue residue of another HA monomer. The generation of the corresponding homodimer occurs through the formation of a disulfide bond, as demonstrated by *in vitro* experiments^7^. Thus, the homodimerization process, by reducing the total circulating pool of free Cys-34, represents another mechanism impairing HA non-oncotic activities, in addition to the well-documented reversible and irreversible oxidation of the Cys-34 residue^25^.

The increased levels of homodimeric isoforms may also explain why the truncated monomers at the C- and N-terminal portion of the molecule (HA-L and HA-DA) do not differ between patients with cirrhosis and healthy controls, as also shown in our previous study when the spectrum of the analysis was restricted to the mass range of the monomeric forms^2^. Indeed, these truncated isoforms may be more prone in patients with cirrhosis to generate homodimers, which can be unveiled only by extending the mass range of the analysis. An experimental confirmation of this hypothesis derives from our *in vitro* experiments in plasma samples exposed to t-BuOOH, which showed a dose-dependent increase of the homodimeric truncated isoforms associated with a mirrored decrease of the corresponding monomeric isoforms. Given these results, it is reasonable to hypothesize that the pro-oxidant and pro-inflammatory vascular microenvironment that characterizes decompensated cirrhosis^4^ is the main driver of this process by promoting the homodimerization of truncated HA isoforms.

This assumption may also explain why the homodimeric, but not the monomeric truncated isoforms, were related with the severity of cirrhosis as assessed prognostic scores, including the CLIF-C OF and the CLIF-C AD scores. This is a further original aspect of the present study, which also explored the relationship between the extent of HA homodimers and some clinical features of liver cirrhosis. Namely, besides the sporadic association between the homodimeric native HA with the occurrence of renal impairment, the relative amount of dimeric hdHA-DA was found consistently higher in patients presenting ascites, renal impairment or bacterial infections at hospital admission, regardless of the type of clinical manifestation. Furthermore, the multivariate analysis unveiled that this association was independent from the severity of the disease.

Given these results, it is not surprising that the relative amount of the HA homodimeric isoforms is significantly increased in patients presenting ACLF at hospital admission. According to the recent systemic inflammation hypothesis^4^, inflammation and oxidative stress are key drivers for acute decompensation and multi-organ dysfunction which may culminate in the ACLF syndrome, a clinical condition associated with a very high short-term mortality rate^26^. By analyzing a subset of patients enrolled in the CANONIC study^26^, it has been shown that the amount of reversible and irreversible oxidized HA at the Cys-34 residue is significantly increased in patients with decompensated cirrhosis and, to a greater extent, in those with ACLF^5^, suggesting that an impaired antioxidant activity of the circulating HA pool may contribute to the development of such syndrome^4^. Our results further support the systemic inflammation hypothesis, as HA homodimers, a fraction of HA with impaired antioxidant activity, are significantly increased in patients with ACLF and their abundance reflect the severity of the syndrome.

Coherent with this finding, a level of hdHA-DA above its best cut-off was able to discriminate patients with a shorter survival. Thus, it would appear that the molecular abnormalities of HA represent an adverse prognostic factor in patients with cirrhosis, an assumption that is reinforced by the finding that the reduction of the relative amount of the monomeric native HA is also associated with a poorer survival, an observation that confirms our previous findings^2^.

Taken together, the results on HA oxidative damage of the present as well as of previous investigations^5^ highlight the relevance of the circulating HA pool as one of the most important mechanism of defense against systemic oxidative stress in decompensated cirrhotic patients. Thus, maintaining an adequate amount of HA molecule with a fully preserved structure and function, the so called “effective albumin concentration”^27^, could represent a future strategy to prevent the development of organ dysfunction and failure in the advanced stage of the disease.

Finally, despite the benefits provided by our platform, our technique presents two limitations that need to be acknowledged. First, beside the three well-characterized dimeric isoforms, some other peaks corresponding to undetermined isoforms were also observed, but they were not considered for the analysis since their very low abundance in both controls and cirrhotic patients. Thus, we cannot exclude the existence of other HA homodimeric isoforms although their relevance is unlikely to be significant. Second, our HPLC-MS does not allow, at present, to quantify the absolute plasma concentrations of the HA isoforms, which, therefore, are expressed as relative abundance.

In conclusion, advanced cirrhosis is characterized by a pro-oxidant and pro-inflammatory status which promotes a wide range of structural damages of the HA molecule. In addition to the already described post-transcriptional changes observed in the monomeric isoforms, the present study demonstrates that homodimerization represents a novel oxidative alteration of the molecule. As a result, HA homodimers can be seen as a novel marker of the circulating redox state in these patients.

Taken together, such post-transcriptional alterations, involving different sites of the molecule, likely lead to different functional consequences, but all bring to a progressive reduction of the native HA, the only isoform provided of structural and functional integrity.

## Additional Information

**How to cite this article**: Baldassarre, M. *et al*. Albumin Homodimers in Patients with Cirrhosis: Clinical and Prognostic Relevance of a Novel Identified Structural Alteration of the Molecule. *Sci. Rep.* **6**, 35987; doi: 10.1038/srep35987 (2016).

**Publisher’s note:** Springer Nature remains neutral with regard to jurisdictional claims in published maps and institutional affiliations.

## Supplementary Material

Supplementary Information

## Figures and Tables

**Table 1:** Clinical characteristics, hemodynamic and biochemical parameters of patients with cirrhosis at study enrolment (n = 123).

**Etiology of cirrhosis**	
Viral	69 (56%)
Alcohol	28 (23%)
Other	26 (21%)
**Complications at hospital admission**	
Ascites	61 (50%)
Bacterial infection	39 (32%)
Renal impairment^1^	30 (24%)
Encephalopathy grade III-IV	12 (10%)
Variceal Bleeding	8 (6%)
HCC^2^ meeting the Milan criteria	13 (10%)
**Hemodynamic and biochemical parameters**	
Mean arterial pressure (mmHg)	81 ± 10
Heart rate (bpm)	73 ± 14
Serum bilirubin (mg/dL)	4.0 ± 5.1
Serum albumin (g/dL)	3.5 ± 0.7
Serum creatinine (mg/dL)	1.2 ± 0.7
Serum sodium (mmol/L)	137 ± 5
INR^3^	1.6 ± 0.4
CRP^4^ (mg/dL)	2.2 ± 3.0
**Prognostic scores**	
Child-Pugh class (A/B/C)	25/72/26 (21/58/21)
Child-Pugh score	8 ± 2
MELD score	17 ± 7
CLIF-C AD	49 ± 8.8
CLIF-C OF	6.8 ± 1.3
ACLF grade (1/2/3)	15/5/1 (12/4/1)
**Comorbidities**	
Diabetes	45 (37%)

Data are presented as mean ± standard deviation or frequencies (%). ^1^Renal impairment = serum creatinine > 1.5 mg/dL; ^2^Hepatocellular carcinoma; ^3^Prothrombin time international normalized ratio; ^4^C-reactive protein.

**Table 2:** Correlations between relative abundance of human albumin (HA) isoforms and MELD, Child-Pugh, CLIF-C OF and CLIF-C AD scores.

	MELD		Child-Pugh		CLIF-C OF		CLIF-C AD	
	*Spearman rho*	*p*	*Spearman rho*	*p*	*Spearman rho*	*p*	*Spearman rho*	*p*
*Homodimeric isoforms*								
hdHA-DA (%)	0.413	**0.001**	0.373	**0.001**	0.186	**0.040**	0.263	**0.005**
hdHA-L (%)	0.369	**0.001**	0.362	**0.001**	0.246	**0.006**	0.253	**0.006**
hdHA (%)	0.539	**0.001**	0.485	**0.001**	0.377	**0.001**	0.434	**0.001**
*Monomeric Isoforms*								
HA-DA (%)	0.117	0.200	0.111	0.226	0.030	0.739	−0.052	0.580
HA-L (%)	−0.186	0.059	−0.169	0.063	−0.079	0.389	−0.162	0.083
Native HA (%)	−0.412	**0.001**	−0.358	**0.001**	−0.240	**0.008**	−0.241	**0.010**

hdHA-DA: homodimeric N-terminal truncated isoform; hdHA-L: homodimeric C-terminal truncated isoform; hdHA: homodimeric native isoform; HA-DA: monomeric N-terminal truncated isoform; HA-L: monomeric C-terminal truncated isoform; HA: monomeric native isoform.
