# Supplementary material for: Albumin Homodimers in Patients with Cirrhosis: Clinical and Prognostic Relevance of a Novel Identified Structural Alteration of the Molecule
Source: Sci Rep. 2016 Oct 26;6:35987. doi: 10.1038/srep35987 (PMC5080612; doi:10.1038/srep35987)
Supplement: Supplementary Information [file srep35987-s1.doc]

**Supplementary informations**

**ALBUMIN HOMODIMERS IN PATIENTS WITH CIRRHOSIS:**

**CLINICAL AND PROGNOSTIC RELEVANCE OF A NOVEL IDENTIFIED STRUCTURAL ALTERATION OF THE MOLECULE.**

Maurizio Baldassarre^1,2^, Marco Domenicali^1,2^, Marina Naldi^3^,

Maristella Laggetta^1,2^, Ferdinando A. Giannone^1,2^, Maurizio Biselli^1^, Daniela Patrono^4^,

Carlo Bertucci^3^, Mauro Bernardi^1,2^, Paolo Caraceni^1,2^.

*^1^Department of Medical and Surgical Sciences, ^2^Center for Applied Biomedical Research (C.R.B.A.), ^3^Department of Pharmacology and Biotechnology,*

*Alma Mater Studiorum University of Bologna, Italy*

*^4^ Centralized Laboratory, S. Orsola-Malpighi University Hospital, Bologna, Italy*

**Supplementary table 1.** *Human albumin (HA) monomeric and homodimeric isoforms relative abundance in cirrhotic patients with or without ascites, renal dysfunction defined as serum creatinine level higher than 1.5mg/dL, or bacterial infection at the time of admission to the hospital.*

|  |  | **Ascites** | | | | | | |  | **Renal impairment** | | | | | | |  | **Bacterial infection** | | | | | | |
| --- | --- | --- | --- | --- | --- | --- | --- | --- | --- | --- | --- | --- | --- | --- | --- | --- | --- | --- | --- | --- | --- | --- | --- | --- |
|  |  | **Yes**  **(N = 61)** | | | **No**  **(N = 62)** | | | ***p*** |  | **Yes**  **(N = 30)** | | | **No**  **(N = 93)** | | | ***p*** |  | **Yes**  **(N = 39)** | | | **No**  **(N = 84)** | | | ***p*** |
| ***Clinical scores*** |  |  |  |  |  |  |  |  |  |  |  |  |  |  |  |  |  |  |  |  |  |  |  |  |
| ***MELD*** |  | 19 | ± | 7 | 15 | ± | 7 | **0.004** |  | 23 | ± | 7 | 15 | ± | 6 | **0.001** |  | 19 | ± | 7 | 16 | ± | 7 | 0.060 |
| ***Child Pugh*** |  | 9 | ± | 1 | 7 | ± | 2 | **0.001** |  | 9 | ± | 2 | 8 | ± | 2 | **0.005** |  | 8 | ± | 1 | 8 | ± | 2 | 0.354 |
| ***Homodimeric isoforms*** |  |  |  |  |  |  |  |  |  |  |  |  |  |  |  |  |  |  |  |  |  |  |  |  |
| **hdHA-DA (%)** |  | 5.4 | ± | 2.0 | 4.3 | ± | 2.0 | **0.002** |  | 5.9 | ± | 2.0 | 4.4 | ± | 1.9 | **0.001** |  | 6.0 | ± | 2.3 | 4.3 | ± | 1.7 | **0.001** |
| **hdHA-L (%)** |  | 2.3 | ± | 1.1 | 2.1 | ± | 1.4 | 0.329 |  | 2.6 | ± | 1.5 | 2.1 | ± | 1.2 | 0.074 |  | 2.5 | ± | 1.2 | 2.1 | ± | 1.3 | 0.065 |
| **hdHA (%)** |  | 3.7 | ± | 4.8 | 2.7 | ± | 2.0 | 0.151 |  | 4.4 | ± | 3.0 | 2.8 | ± | 3.9 | **0.036** |  | 3.6 | ± | 2.2 | 2.9 | ± | 4.2 | 0.245 |
| ***Monomeric isoforms*** |  |  |  |  |  |  |  |  |  |  |  |  |  |  |  |  |  |  |  |  |  |  |  |  |
| **HA-DA (%)** |  | 4.7 | ± | 1.9 | 4.0 | ± | 2.6 | 0.070 |  | 4.55 | ± | 2.1 | 4.3 | ± | 2.4 | 0.560 |  | 4.6 | ± | 2.1 | 4.2 | ± | 2.4 | 0.427 |
| **HA-L (%)** |  | 4.8 | ± | 8.2 | 4.2 | ± | 3.2 | 0.575 |  | 3.5 | ± | 2.0 | 4.8 | ± | 7.0 | 0.310 |  | 3.5 | ± | 1.8 | 4.9 | ± | 7.4 | 0.227 |
| **Native HA (%)** |  | 79.0 | ± | 9.2 | 82.7 | ± | 5.3 | **0.007** |  | 79.1 | ± | 4.6 | 81.6 | ± | 8.3 | **0.042** |  | 79.7 | ± | 5.0 | 81.4 | ± | 8.6 | 0.175 |

hdHA-DA: homodimeric N-terminal truncated isoform; hdHA-L: homodimeric C-terminal truncated isoform; hdHA: homodimeric native isoform; HA-DA: monomeric N-terminal truncated isoform; HA-L: monomeric C-terminal truncated isoform; HA: monomeric native isoform.

***Supplementary table 2.*** *Backward multivariate binary logistic regression analysis of parameters associated to the presence of ascites, renal dysfunction defined as serum creatinine level higher than 1.5mg/dL, or bacterial infection at the time of admission to the hospital.*

|  |  | **Covariates** | **β** | **SD** | **OR (95%CI)** | ***p*** |
| --- | --- | --- | --- | --- | --- | --- |
| **Ascites** | *STEP 1* | Native HA | -0.037 | 0.035 | 0.963 (0.899-1.033) | 0.291 |
|  |  | hdHA-DA | 0.224 | 0.122 | 1.251 (0.984-1.589) | 0.067 |
|  |  | MELD | 0.050 | 0.030 | 1.051 (0.992-1.114) | 0.090 |
|  | *STEP 2* | **hdHA-DA** | **0.279** | **0.107** | **1.321 (1.071-1.630)** | **0.009** |
|  |  | MELD | 0.056 | 0.030 | 1.057 (0.998-1.120) | 0.059 |
| **Renal impairment** | *STEP 1* | Native HA | 0.050 | 0.064 | 1.051 (0.926-1.192) | 0.440 |
|  |  | hdHA-DA | 0.386 | 0.174 | 1.471 (1.045-2.071) | 0.027 |
|  |  | hdHA | 0.068 | 0.071 | 1.070 (0.932-1.229) | 0.336 |
|  |  | Child Pugh | 0.244 | 0.141 | 1.276 (0.968-1.682) | 0.084 |
|  | *STEP 2* | hdHA-DA | 0.303 | 0.125 | 1.354 (1.062-1.727) | 0.014 |
|  |  | hdHA | 0.039 | 0.062 | 1.040 (0.922-1.173) | 0.527 |
|  |  | Child Pugh | 0.228 | 0.138 | 1.256 (0.982-1.645) | 0.098 |
|  | *STEP 3* | **hdHA-DA** | **0.320** | **0.122** | **1.377 (1.084-1.750)** | **0.009** |
|  |  | Child Pugh | 0.246 | 0.135 | 1.279 (0.982-1.665) | 0.068 |
| **Bacterial infection** | *STEP 1* | hdHA-DA | 0.405 | 0.119 | 1.499 (1.187-1.893) | 0.001 |
|  |  | MELD | 0.020 | 0.029 | 1.020 (0.963-1.080) | 0.504 |
|  | *STEP 2* | **hdHA-DA** | **0.426** | **0.115** | **1.537 (1.229-1.922)** | **<0.001** |

**Supplementary table 3.** *Human albumin (HA) monomeric and homodimeric isoforms relative abundance in cirrhotic patients with or without variceal bleeding or grade III and IV hepatic encephalopathy at the time of admission to the hospital.*

|  |  | **Variceal bleeding** | | | | | | |  | **Hepatic encephalopathy** | | | | | | |
| --- | --- | --- | --- | --- | --- | --- | --- | --- | --- | --- | --- | --- | --- | --- | --- | --- |
|  |  | **Yes**  **(N = 8)** | | | **No**  **(N = 115)** | | | ***p*** |  | **Yes**  **(N = 12)** | | | **No**  **(N = 111)** | | | ***p*** |
| ***Homodimeric isoforms*** |  |  |  |  |  |  |  |  |  |  |  |  |  |  |  |  |
| **hdHA-DA (%)** |  | 4.0 | ± | 1.6 | 4.9 | ± | 2.1 | 0.217 |  | 5.5 | ± | 2.2 | 4.8 | ± | 2.0 | 0.221 |
| **hdHA-L (%)** |  | 1.6 | ± | 1.4 | 2.3 | ± | 1.3 | 0.145 |  | 2.9 | ± | 1.8 | 2.2 | ± | 1.2 | 0.207 |
| **hdHA (%)** |  | 2.0 | ± | 2.2 | 3.2 | ± | 3.8 | 0.374 |  | 4.3 | ± | 4.3 | 3.0 | ± | 3.6 | 0.286 |
| ***Monomeric isoforms*** |  |  |  |  |  |  |  |  |  |  |  |  |  |  |  |  |
| **HA-DA (%)** |  | 4.1 | ± | 1.9 | 4.4 | ± | 2.3 | 0.791 |  | 4.2 | ± | 1.7 | 4.4 | ± | 2.4 | 0.827 |
| **HA-L (%)** |  | 3.9 | ± | 2.1 | 4.5 | ± | 6.4 | 0.790 |  | 3.5 | ± | 1.5 | 4.6 | ± | 6.5 | 0.578 |
| **Native HA (%)** |  | 84.3 | ± | 4.9 | 80.7 | ± | 7.8 | 0.192 |  | 79.6 | ± | 4.8 | 81.0 | ± | 8.0 | 0.528 |

hdHA-DA: homodimeric N-terminal truncated isoform; hdHA-L: homodimeric C-terminal truncated isoform; hdHA: homodimeric native isoform; HA-DA: monomeric N-terminal truncated isoform; HA-L: monomeric C-terminal truncated isoform; HA: monomeric native isoform.
